# Formation of Akaganeite in Atmospheric Corrosion of Carbon Steel Induced by NaCl Particles in an 85% RH Environment

**DOI:** 10.3390/ma18194462

**Published:** 2025-09-24

**Authors:** Haigang Xiao, Hongbo Zhang, Yan Guo, Hongduo Hao, Hao Chang, Ying Li

**Affiliations:** 1 Xi’an Thermal Power Research Institute Co., Ltd., Xi’an 710032, China; zhanghongbo@tpri.com.cn (H.Z.); guoyan@tpri.com.cn (Y.G.); haohongduo@tpri.com.cn (H.H.); changhao@tpri.com.cn (H.C.); 2 Institute of Metal Research, Chinese Academy of Sciences, Shenyang 110016, China; liying@imr.ac.cn

**Keywords:** akaganeite, atmospheric corrosion, salt

## Abstract

Akaganeite is the most destructive corrosion product in a rust layer, and it accelerates the corrosion rate of steel in certain atmospheres. Until now, considerable controversy has existed regarding the conditions required for its formation and its mechanism of formation. In this work, the formation of akaganeite in the specific corrosion process, which was atmospheric corrosion induced by NaCl deliquescence, was investigated through simulated experiments in a laboratory setting. Stereoscopic microscopy and scanning electron microscopy were employed to characterize the morphologies of the corrosion products, which could illuminate the morphological features of the electrolyte induced by the NaCl particles. The constituents of rust in a single droplet were analyzed by micro-Raman spectroscopy, and the components of the corrosion phases on a macroscopic scale were analyzed by XRD. The results indicate that the deliquescence of NaCl particles caused droplets to form around them, and atmospheric corrosion occurred in each droplet independently. Akaganeite can form during atmospheric corrosion induced by NaCl particles in the early stage within 12 h. The initial corrosion products, lepidocrocite and magnetite, increase the amount of akaganeite formed. The amount of salt deposited also plays an essential role in the formation of akaganeite on a macroscopic scale.

## 1. Introduction

Steel exposed to marine atmospheres suffers from severe atmospheric corrosion [1,2,3,4,5,6,7]. The composition of a rust layer determines its protective ability [6,7,8,9], and specifically, the presence of akaganeite (β-FeOOH) in the rust layer is considered one of the essential reasons for deteriorative corrosion [10,11,12,13,14]. However, the conditions required for its formation and its mechanism of formation in atmospheric corrosion have not yet been clarified [15,16]. Atmospheric corrosion occurs on metals within the cover of an electrolyte layer, which is an electrochemical corrosion process [17,18,19]. Electrolytes can form on or arrive at a metal surface by two main modes, direct deposition and sorption [20]. The formation mode of the electrolyte layer determines the features of the liquid film and subsequently influences the formation process of the corrosion products. Due to the high levels of airborne NaCl and humidity in marine atmospheres, atmospheric corrosion in these environments is frequently induced by the deliquescence of salt deposited on the surface of steel, which is a type of sorption. Thus, our investigation of the formation process and conditions of akaganeite formation in this specific process could not only elucidate the mechanism of akaganeite formation but also allow great advances in corrosion protection methods for steel.

Deliquescence is the absorption of water vapor from a moist environment by a soluble salt to generate a liquid electrolyte. It occurs because the vapor pressure of a saturated aqueous salt solution is less than the vapor pressure of pure water [21]. Deliquescence occurs at a specific relative humidity, which is referred to as the deliquescence relative humidity, and for NaCl, that humidity is 75%. J. Weissenrieder [22] observed the fast deliquescence of NaCl crystallites at high relative humidity, and corrosion began in the droplets containing NaCl solution. Shengxi Li [23] investigated the effect of salt crystal size on atmospheric corrosion and suggested that only NaCl particles greater than 45–100 μm in diameter induced corrosion. Chunling Li [24] characterized the morphologies of corrosion products formed on weathering steel from deposited salt exposed to high-humidity environments by SEM and found that the corrosion products were essentially nodular. According to previous studies, a droplet forms around the NaCl particle in humid environments, and corrosion occurs under the droplet. Compared to the complete steel surface, the electrolyte formed by deliquescence is discontinuous and corrosion reactions proceed independently in every isolated droplet. In contrast, during direct deposition, a continuous and thick liquid film forms on the metal surface but dries out under certain environment conditions. Thus, trends in the changes in the chloride concentration and the thickness of the electrolyte layer are different in the corrosion processes induced by different electrolyte formation modes. Furthermore, according to previous studies on the conditions of akaganeite formation, sufficiently high chloride concentration [15,25,26] and oxygen supply throughout the thin liquid film to oxidize green rust [13,26,27,28] are required for akaganeite formation. Therefore, the formation conditions for akaganeite may be different in the two modes, and investigation of the akaganeite formation process during NaCl particle-induced atmospheric corrosion is necessary.

Until now, very few studies focused on the constituents of the corrosion products formed during NaCl particle-induced atmospheric corrosion have been published. Shengxi Li [29] identified, for the first time, the corrosion products formed on carbon steel under NaCl droplets that formed through the deliquescence of pre-deposited NaCl particles upon exposure to high humidity. They found that corrosion began under the NaCl droplets, and then lepidocrocite (γ-FeOOH) and a type of green rust formed first. This green rust was converted to lepidocrocite and magnetite (Fe_3_O_4_) as corrosion progressed. J. Forsberg [30] utilized time-resolved in situ X-ray spectroscopy to investigate the initial stage of atmospheric corrosion induced by deposited salt and detected a chloride-containing phase. To the best of our knowledge, the formation of akaganeite during atmospheric corrosion induced by NaCl deliquescence has not been reported previously.

Considering the factors mentioned above, the formation of akaganeite in NaCl particle-induced atmospheric corrosion has not been studied, although those conditions may be important for its formation. Additionally, the parameters that influenced this process have not been illuminated.

In the present work, the formation mechanism of akaganeite during the deliquescence process of salt deposits on steel surfaces was investigated. Samples with pre-deposited salt were exposed to a high-humidity environment (85%) to simulate the atmospheric corrosion process induced by the deliquescence of NaCl particles. Stereoscopic microscopy and SEM were employed to characterize the morphological features of the corrosion products formed in these samples. The constituents of rust in a single droplet were analyzed by micro-Raman spectroscopy, and the components of the corrosion phases over a macroscopic region were analyzed by XRD. Through the in situ Raman spectroscopy, the formation process of corrosion products was detected during the early stages of corrosion (within 12 h). The parameters that influenced the formation of akaganeite during deliquescence were investigated by analyzing the results of simulated experiments carried out under different parameters. Furthermore, the role of initial corrosion products in the formation of akaganeite during deliquescence was also discussed.

## 2. Materials and Methods

### 2.1. Material

Samples of carbon steel Q235, which is square-shaped, with dimensions of 10 mm, 15 mm, and 5 mm, were employed in this work. The composition (wt. %) of Q235 includes C (0.176), S (0.023), P (0.019), Mn (0.57), Si (0.233), and Cu (0.033), with Fe composing the remainder. The samples were wet-polished to 1000-grade emery paper, cleaned with 95% ethanol under ultrasonication, rinsed with distilled water, dried, and stored in a moisture-free desiccator prior to use. The corrosion solution was prepared from analytically pure sodium chloride and distilled water.

### 2.2. Characterization of the Iron Rust Phases

To investigate the morphological features of the corrosion products formed during atmospheric corrosion induced by NaCl deliquescence, optical and SEM analyses were carried out. A Zeiss Stemi 508 (Zeiss, Jena, Germany) equipped with an Axiocam 105 Color microscopic camera (Zeiss, Jena, Germany) was utilized to obtain optical micrographs of the corrosion products formed on the samples after corrosion tests at 6.3 times and 50 times magnification. Microscopic observations were carried out using an SEM (INSPECT F50) (FEI, Eindhoven, The Netherlands) equipped with a secondary electron detector and energy-dispersive X-ray diffraction (EDX) (FEI, Eindhoven, The Netherlands). The acceleration voltage for SEM-EDX experiments was 20 kV.

Micro-Raman spectroscopy was used to analyze the constituents of the corrosion products on a microscopic scale. Micro-Raman analysis was performed on a LabRam HR800 (Horiba) Raman spectrometer (Villeneuve-d’Ascq, France)with a He-Ne gas laser (excitation wavelength 632.8 nm) coupled to a Leica microscope (Leica, Wetzlar, Germany) to focus the beam on a 1 μm diameter area, and the optical video microscope allowed for simultaneous optical imaging during Raman measurements. Raman spectra were collected in a backscattering geometry. The laser power focused through a 50× objective lens with a numerical aperture of 0.5. A 200 μm aperture and 1800 g/mm diffraction grating were used in the system. The maximum laser power was 25 mW. Because iron-containing compounds can be heated with focused laser radiation, neutral laser density filter D1 was used in order to reduce the laser power by 10 times. Thus, the laser power was 2.5 mW. A total accumulation time of 30 s was used.

Since XRD analysis allows to obtain information from corrosion products in an approximately 1 mm × 10 mm area, this technique was performed directly on the rusted steel plate to characterize the rust phases on a macroscopic scale. XRD measurements were performed with a Rigaku-D/max 2500 PC diffractometer (Rigaku, Tokyo, Japan) equipped with a Cu X-ray tube. A current of 300 mA and a voltage of 50 kV were set as the tube settings. The XRD data were collected over a 2θ range of 10° to 40° with a step size of 0.02° because the characteristic diffraction peaks for the relevant phases are in this range. The crystalline phases present in the rust formed on steels were identified from XRD patterns using the JCPDS database (Corrosion Phases 65/24988*) and Jade 5.0 software (Version number 5.0).

### 2.3. The Simulated Experiment Procedures

The salt particles were deposited on the sample surfaces by dispersing sodium chloride solution and evaporating the solvent. To enhance the spreadability and evaporation rate of the solution, a mixture of 50% alcohol and 50% NaCl solution was employed to wet the surfaces of the samples. After wetting the surface with 0.1 mL of the alcohol/NaCl mixture by pipette, the samples were dried in an oven at 60 °C. After ten minutes, the solvent evaporated, and crystalline salt was deposited on the surface of the steel. The amount of salt deposited was controlled by the concentration of NaCl in the solution. Considering that the salinity in real marine atmospheres can reach 1500 mg m^−2^d^−1^ according to ISO 9223 [31], the amounts of deposited salt in this study were 194.8 mg/m^2^ and 1948 mg/m^2^, and the concentrations of NaCl solution employed were 0.01 mol/L and 0.1 mol/L.

The samples with pre-deposited salt particles were exposed to an environment at 30 °C and 85% RH, and the conditions were maintained by a constant temperature and humidity test chamber (LRHS-101-LH) manufactured by Shanghai Linpin Instruments Co. Ltd. (Shanghai, China). After exposure to the high-humidity environment for 12 h and 24 h, samples were removed from the chamber and dried by cold wind to halt the corrosion reaction. Then, the corrosion products formed on the surfaces of the samples were characterized by each analysis technique.

## 3. Results and Discussion

### 3.1. Morphological Features of the Electrolytes Formed During Atmospheric Corrosion Induced by NaCl Deliquescence

**Figure 1:**
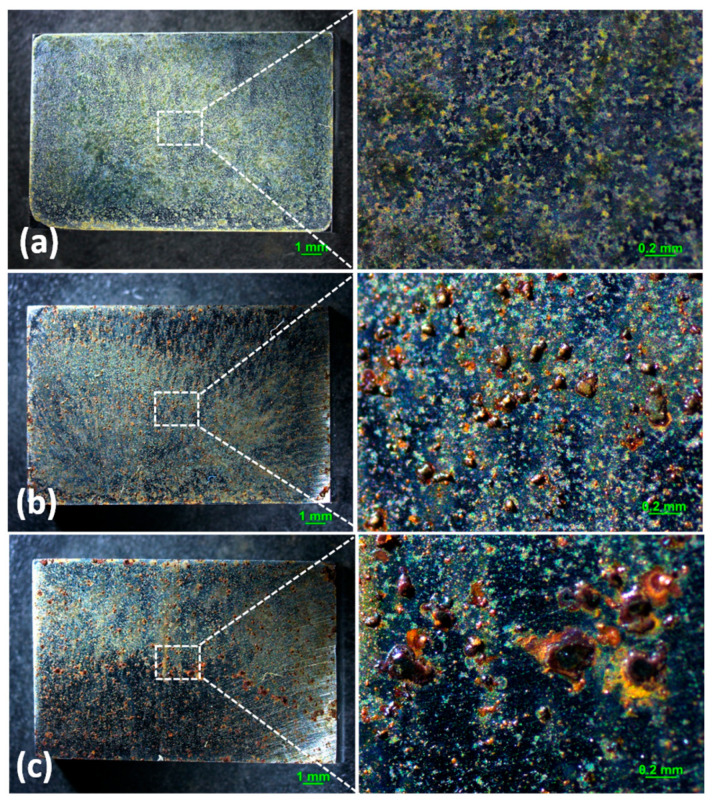
Optical images of the morphology of (**a**) samples with 194.8 mg/m^2^ of deposited salt, (**b**) samples with 194.8 mg/m^2^ of deposited salt exposed to high humidity for 12 h, and (**c**) samples with 194.8 mg/m^2^ of deposited salt exposed to high humidity for 24 h.

**Figure 2:**
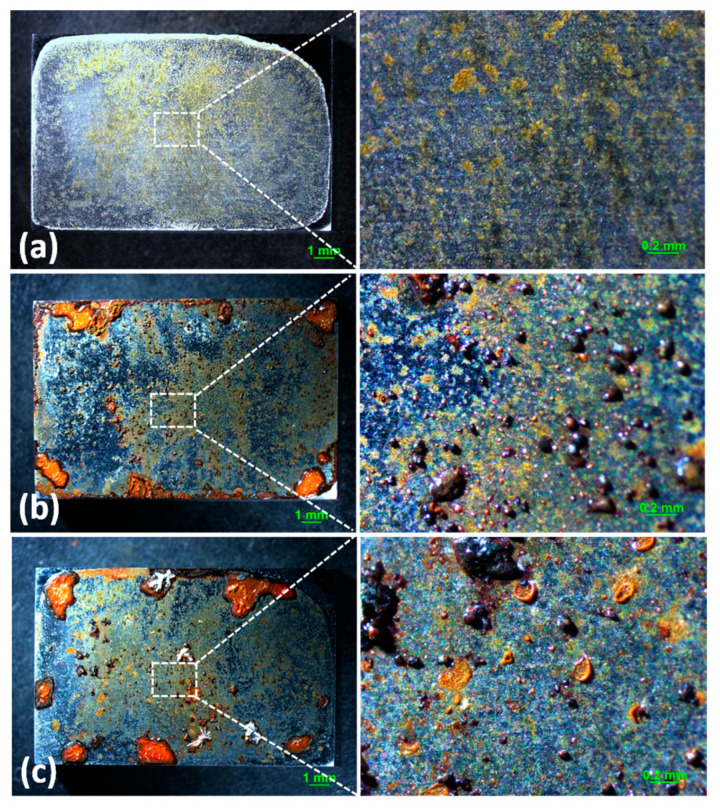
Optical images of the morphology of (**a**) samples with 1948 mg/m^2^ of deposited salt, (**b**) samples with 1948 mg/m^2^ of deposited salt exposed to high humidity for 12 h, and (**c**) samples with 1948 mg/m^2^ of deposited salt exposed to high humidity for 24 h.

Optical microscopy images of the corrosion products formed on the surface with 194.8 mg/m^2^ of deposited NaCl after different periods during atmospheric corrosion induced by NaCl particle deliquescence are shown in Figure 1. Figure 1a shows the surface of samples after 194.8 mg/m^2^ of salt was deposited. The images suggest some corrosion products formed during the salt deposition process, but the surface of the samples was flat and did not bulge. As shown in Figure 1b, discontinuous and nodular corrosion products formed on the surface of the metals, but regions without droplets were clean after 12 h of exposure to a high-humidity environment. After 24 h, corrosion products were distributed discontinuously on the metal surface. Figure 2 shows the optical observation of the corrosion results for samples with 1948 mg/m^2^ of deposited NaCl after different periods of exposure during atmospheric corrosion induced by NaCl particle deliquescence. The morphologies of the corrosion products in these samples were similar to those of the samples with 194.8 mg/m^2^ of NaCl.

**Figure 3:**
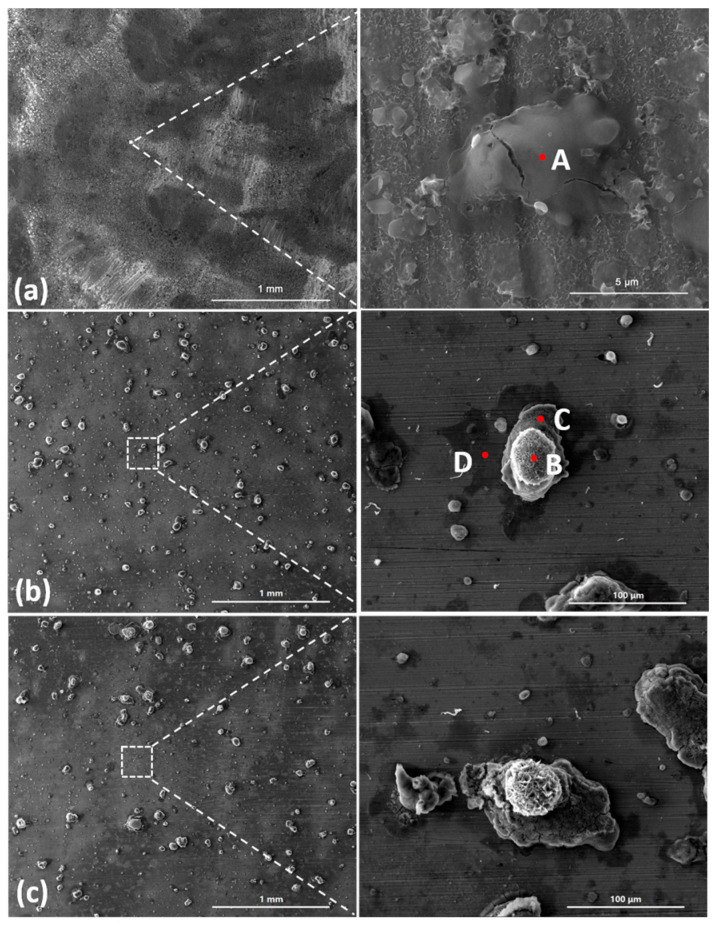
SEM micrographs of (**a**) samples with 194.8 mg/m^2^ of deposited salt, (**b**) samples with 194.8 mg/m^2^ of deposited salt exposed to high humidity for 12 h, and (**c**) samples with 194.8 mg/m^2^ of deposited salt exposed to high humidity for 24 h.

**Figure 4:**
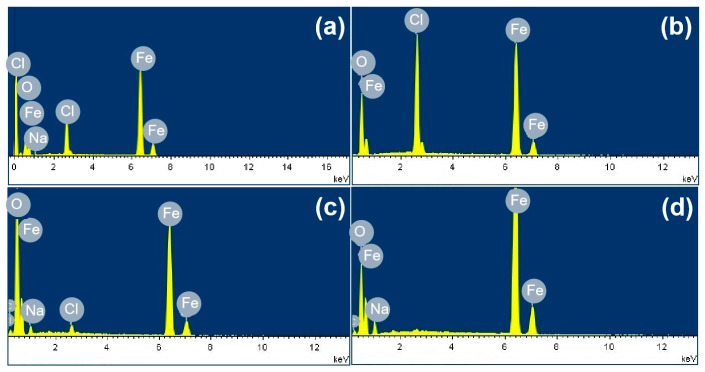
Elemental analysis of the rust layer: (**a**) elemental distribution at point A in Figure 3a, (**b**) elemental distribution at point B in Figure 3b, (**c**) elemental distribution at point C in Figure 3b, and (**d**) elemental distribution at point D in Figure 3b.

**Figure 5:**
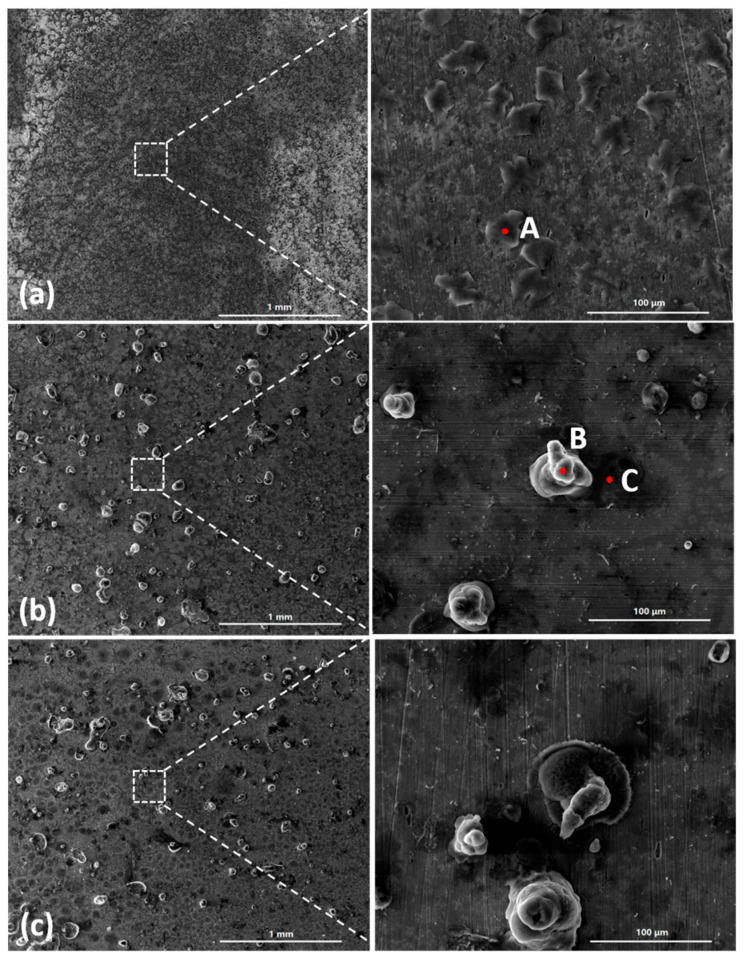
SEM micrographs of (**a**) samples with 1948 mg/m^2^ of deposited salt, (**b**) samples with 1948 mg/m^2^ of deposited salt exposed to high humidity for 12 h, and (**c**) samples with 1948 mg/m^2^ of deposited salt exposed to high humidity for 24 h.

**Figure 6:**
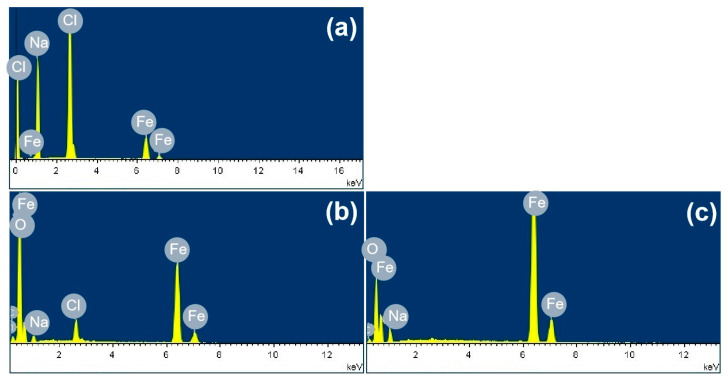
Elemental analysis of the rust layer: (**a**) elemental distribution at point A in Figure 5a, (**b**) elemental distribution at point B in Figure 5b, and (**c**) elemental distribution at point C in Figure 5b.

Figure 3 shows the SEM images of the corrosion products formed after different durations of atmospheric corrosion induced by NaCl particle deliquescence. The EDS results presented in Figure 4 show that NaCl particles were detected on the surface of the samples after the salt deposition process. After 12 h of exposure to high humidity, the locations with pre-deposited salt particles had corroded. The edges of the corrosion products were round, and the corrosion products on the steel surfaces were clustered spheres. The morphologies of the corrosion products in simulated experiments confirmed that crystalline NaCl dissolved in droplets in high humidity due to deliquescence. Corrosion began in each droplet. Figure 5 shows the SEM images of samples with 1948 mg/m^2^ pre-deposited salt, which are similar to those with 194.8 mg/m^2^ of NaCl. The EDS results are presented in Figure 6.

Based on the SEM observations of samples with NaCl particles and relevant EDS results, the NaCl particles deposited on the surfaces of the metals after evaporation of the NaCl solution were isolated. When the relative humidity of the surrounding environment exceeded the deliquescence point, droplets formed around the NaCl particles due to deliquescence. Corrosion occurred, and corrosion products formed in each droplet. Therefore, after the solvent was evaporated from the electrolyte, the corrosion products were spherical, and clusters were observed. The optical microscopy images and SEM observations also demonstrated that the electrolytes formed during atmospheric corrosion induced by NaCl deliquescence were discontinuously dispersed on the surfaces. In the atmospheric corrosion process induced by precipitation, a continuous electrolyte film formed on the surface of the metals, and the soluble substances could migrate into the solution. As a result, corrosion products formed on the whole surface of the metal as a rust layer. However, the corrosion process in each droplet induced by the deliquescence of NaCl is independent and was not influenced by other droplets. The morphological features of the electrolyte are different from those of atmospheric corrosion induced by precipitation [32].

The morphological features of the electrolyte can influence the corrosion process. On one hand, the features affect the concentration of chloride in the solution. During deliquescence, a small quantity of water forms around a salt particle, and a concentrated solution film forms. The driving force for deliquescence is the difference between the vapor pressure of a saturated aqueous salt solution and the vapor pressure of pure water. As the droplet begins to form, the droplet is saturated with chloride. As deliquescence progresses, more water joins the droplet, and the concentration of chloride decreases. Thus, the magnitude of the driving force decreased as the concentration of NaCl in the solution decreased. Consequently, the volume of a stable droplet depends on the amount of NaCl particles pre-deposited and the relative humidity of the environment. Initially, the volume of the electrolyte is limited, and the concentration of chloride stays relatively high in the droplet. Corrosion reactions occurred in each droplet because they were small and rich in chloride. On the other hand, the small volume of droplets also guarantees sufficient oxygen supply from the surrounding environments during the corrosion process. Therefore, the morphology of the electrolyte induced by NaCl particles makes corrosion in an environment with a high concentration of chloride and a sufficient oxygen supply possible [32].

### 3.2. Constituents of Corrosion Products Formed in Single Droplet

As discussed in the previous section, atmospheric corrosion induced by NaCl particle deliquescence produced a large number of isolated corrosion regions, which were micrometers in size. To characterize the constituents of the corrosion products formed in single droplets during this process, micro-Raman analysis was conducted. Since some corrosion products formed during the NaCl deposition process, the constituents of these phases were studied in advance. Then, the newly generated products could be determined by comparing the variations in the constituents during the deliquescence of NaCl particles.

**Figure 7:**
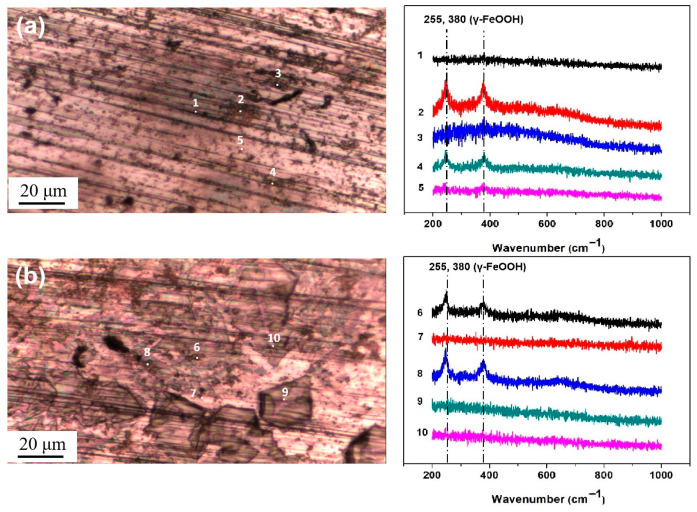
Micro-Raman spectra from different locations on the surface of the steel after salt deposition: (**a**) 194.8 mg/m^2^; (**b**) 1948 mg/m^2^.

Figure 7 shows the optical images of the surface of the steel with 194.8 mg/m^2^ of salt deposited and the micro-Raman analysis results for five representative points. The Raman spectra are named according to the number of points labeled on the optical images. From the comparison with the reference spectra of iron oxyhydroxides and oxides in the references [33,34,35,36], the peaks in points 2 and 4 were found to correspond to those of lepidocrocite (which is considered to be the initial corrosion product) at 255 and 380 cm^−1^, which was also detected in [37,38]. In points 1, 3, and 5, no obvious corrosion products were detected. Similar results were observed on the surface with 1948 mg/m^2^, lepidocrocite was detected at points 6 and 8, and no products were formed at the other points. Lepidocrocite was the only corrosion product formed during the salt deposition process. No akaganeite was obtained on the metal surfaces during the salt deposition process.

**Figure 8:**
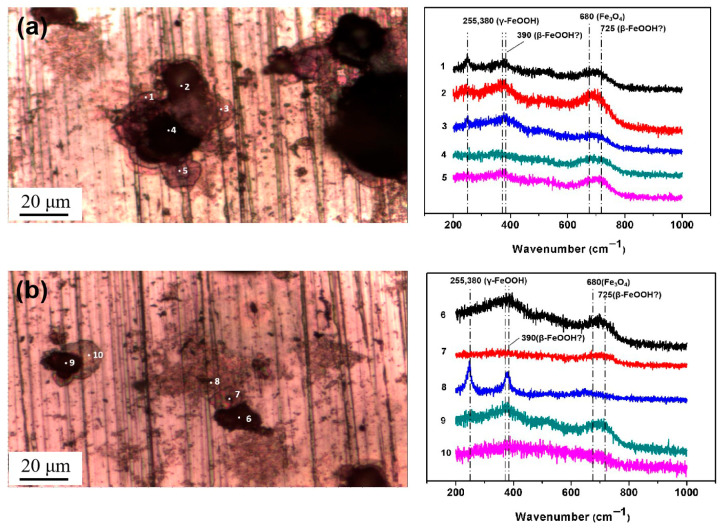
Micro-Raman spectra from different locations on the surface of the steel with 194.8 mg/m^2^ of deposited salt that was exposed to a high-humidity environment for 12 h. (**a**) Position 1; (**b**) Position 2.

**Figure 9:**
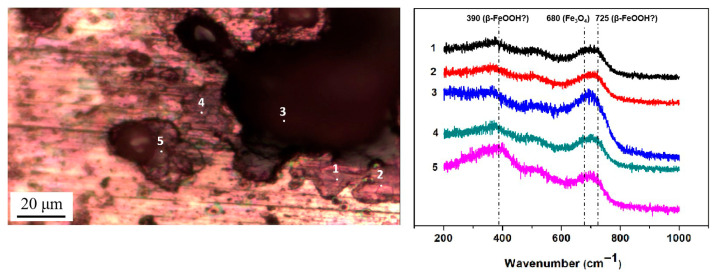
Micro-Raman spectra from different locations on the surface of steel with 1948 mg/m^2^ of deposited salt that was exposed to a high-humidity environment for 12 h.

Then, micro-Raman analysis was carried out on the sample that had been exposed to an environment with 85% RH for 12 h with 194.8 mg/m^2^ of pre-deposited salt. Figure 8a,b display a cluster of corrosion products on the steel surface, with the Raman spectra from the different points labeled in the optical image. After 12 h of exposure in a high-humidity environment, newly formed peaks were detected via Raman spectroscopy at several locations, notably at 390 cm^−1^, 680 cm^−1^, and 725 cm^−1^. According to references [33,34,35,36], the peak at 680 cm^−1^ in Figure 8a,b corresponds to magnetite. Referring to multiple literature sources [33,34,35,36], the characteristic Raman peaks of akaganeite typically occur around 300 cm^−1^, 387 cm^−1^, 413 cm^−1^, and 725 cm^−1^. Therefore, we speculate that the peaks detected at 390 cm^−1^ and 725 cm^−1^ may be attributed to the formation of akaganeite. Although the peak around 300 cm^−1^ is not observed in Figure 8a,b, its absence may be due to the strong absorption of laser radiation by magnetite or the small amount of akaganeite itself. At points 2, 3, 5, 6, and 9, based on the position of the newly added peak, it is speculated that akaganeite may have formed. In addition, akaganeite was absent in the peripheral region. Figure 9 displays the surface with 1948 mg/m^2^ of salt deposited and the associated Raman spectra. In this case, magnetite was detected at all five points, and akaganeite may have also formed.

The results indicate that new corrosion products formed on the carbon steel surface under both salt deposition conditions (194.8 mg/m^2^ and 1948 mg/m^2^) after the deposited salt underwent hydrolysis in a high-humidity environment. Although spectral peaks potentially corresponding to akaganeite were detected at specific locations, its presence could not be conclusively confirmed due to a low concentration or interference from other compounds.

### 3.3. Parameters That Influenced the Formation of Akaganeite During Deliquescence

In the laboratory experiments, some initial corrosion products formed during the salt deposition process. The influence of the initial atmospheric corrosion products was investigated by comparison to the laboratory experiments. In group one, samples were immersed in 0.3 mol/L NaCl solution for 12 h prior to the formation of the initial corrosion products on their surface. The samples in this group were referred to as group one. In group two, samples without this treatment were employed. All these samples were subjected to the salt deposition process and exposed to a high-humidity environment for 12 h. The samples in this group were referred to as group two. Meanwhile, to analyze the effects of salt deposition, 194.8 mg/m^2^ and 1948 mg/m^2^ of salt were deposited in each group.

**Figure 10:**
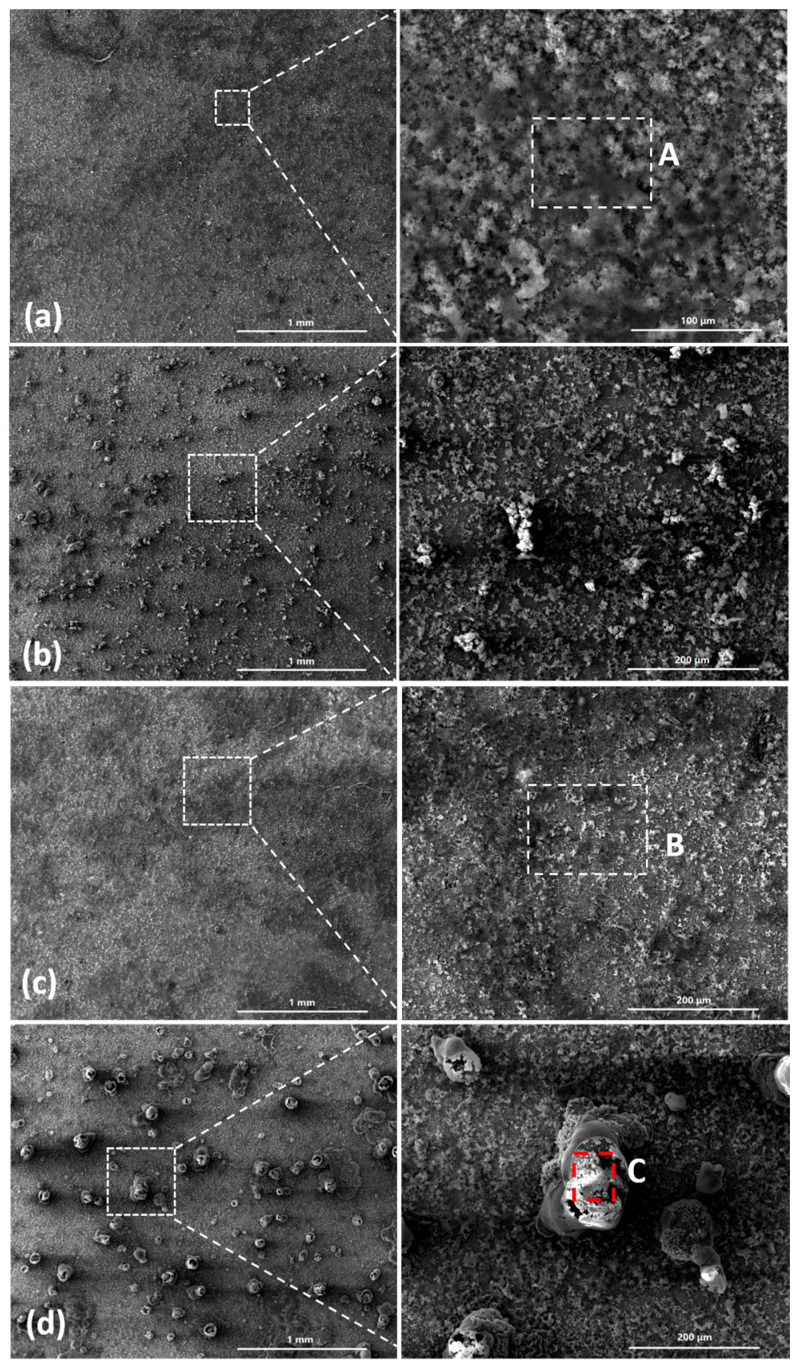
SEM observations of (**a**) samples with initial corrosion products and 194.8 mg/m^2^ of deposited salt; (**b**) samples with initial corrosion products and 194.8 mg/m^2^ of deposited salt exposed to an RH = 85% environment for 12 h; (**c**) samples with initial corrosion products and 1948 mg/m^2^ of deposited salt; and (**d**) samples with initial corrosion products and 1948 mg/m^2^ of deposited salt exposed to an RH = 85% environment for 12 h.

**Figure 11:**
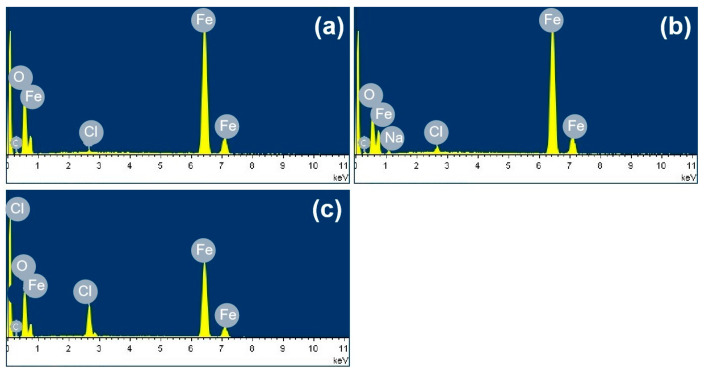
Elemental analysis of the rust layer: (**a**) elemental distribution of area A in Figure 10a, (**b**) elemental distribution of area B in Figure 10c, and (**c**) elemental distribution of area C in Figure 10d.

The SEM observations of the corrosion products formed on the group one substrates are displayed in Figure 10. Figure 10a,c show the initial morphologies of the surfaces after 194.8 mg/m^2^ and 1948 mg/m^2^ of salt had been deposited, respectively. Figure 10b,d depict the morphologies of the corrosion products formed during the deliquescence process. The EDS results are presented in Figure 11. After 12 h, raised corrosion products were formed on the steel surface, which indicated the formation of droplets due to deliquescence. The sizes of the raised corrosion products formed on the samples with initial corrosion products were larger than those on the samples without initial corrosion products.

**Figure 12:**
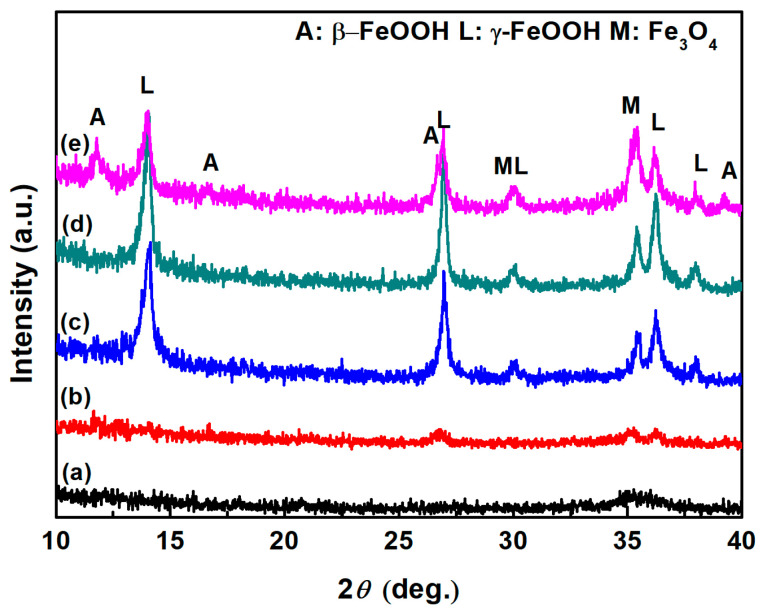
XRD patterns of the rust layer formed on (a) samples with 194.8 mg/m^2^ of deposited salt exposed to high humidity for 12 h, (b) samples with 1948 mg/m^2^ of deposited salt exposed to high humidity for 12 h, (c) samples with initial corrosion products formed by the pretreatment, (d) pretreated samples with 194.8 mg/m^2^ of deposited salt exposed to high humidity for 12 h, and (e) pretreated samples with 1948 mg/m^2^ of deposited salt exposed to high humidity for 12 h.

In the previous section, the micro-Raman results suggest the possible presence of a small amount of akaganeite in each droplet. The relative amount of akaganeite was correlated with the corrosion rate [14]. Thus, it is necessary to macroscopically characterize the constituents of corrosion products. Figure 12 shows the XRD patterns of samples with different initial conditions. To compare the amount of akaganeite formed over a large area, XRD was utilized to characterize the constituents of corrosion. This technique collected the diffraction information in an area approximately 1 mm × 10 mm in size. Compared to micro-Raman spectroscopy, XRD could elucidate the constituents of the corrosion products macroscopically. The top three XRD peaks for akaganeite are 11.840°, 26.627°, and 35.150° according to JCPDS (042-1315). The top three XRD peaks for magnetite are 30.095°, 35.422°, and 62.515° according to JCPDS (019-0629). The top three XRD peaks for lepidocrocite are 14.136°, 27.080°, and 36.342° according to JCPDS (008-0098) [32]. Figure 12a,b show the XRD patterns of samples without initial corrosion products with salt amounts of 194.8 mg/m^2^ and 1948 mg/m^2^ being deposited, respectively. However, the intensity of the diffraction peaks was too low to assign to any specific corrosion products. In group one, to clarify the phases newly generated during deliquescence, the initial corrosion products formed by pretreatment were characterized by XRD prior to exposure to the high-humidity environment. In addition, the constituents of the initial corrosion products formed by the pretreatment were determined to be lepidocrocite and magnetite, as shown in Figure 12c. After 12 h of exposure to the high-humidity environment, the components of the samples with 194.8 mg/m^2^ of salt did not change, and no peak indicative of akaganeite appeared. The identities of the constituents were not determined from XRD analysis due to the small amount of each constituent present. However, akaganeite was clearly formed on the macroscopic scale on the sample with 1948 mg/m^2^ of deposited salt.

Combined with the test results of the micro-Raman analysis and XRD, the results demonstrated that akaganeite could form during the deliquescence of NaCl. High concentrations of chloride and Fe^2+^ are necessary for the formation of akaganeite [25]. Since akaganeite is a product of green rust [32], oxygen is also required as an oxidant. In each droplet, the Fe^2+^ generated by the corrosion reactions accumulated, and its concentration became quite high since the droplets were isolated and substances could not migrate. The high concentrations of oxygen and chloride satisfy the required conditions for akaganeite formation, and akaganeite was therefore formed.

In previous studies, researchers [13] suggested that akaganeite is formed during the dry periods of wet–dry cycles, and it is the product of a type of green rust. In the drying process, as the electrolyte evaporates, the concentration of chloride in the liquid increases. Additionally, the reduction in the thickness of the liquid film was beneficial for the transportation of oxygen. Both of these changes promote the formation of akaganeite. In atmospheric corrosion induced by deliquescence, the conditions are similar to those of the drying process in wet–dry cycles. The results of this study proved that akaganeite can form during atmospheric corrosion induced by deliquescence during the early stage of corrosion (within 12 h). For the formation of akaganeite in cases with a continuous electrolyte film, an excess of salt deposited per area above the critical value was essential to guarantee the requisite high concentration of chloride in the solution. However, in atmospheric corrosion induced by deliquescence, corrosion reactions occurred in each droplet. The volumes of the droplets were correlated with the size of the NaCl particles. As a result, the concentration of chloride in each droplet was high regardless of the amount of salt deposited on the steel surface. In each droplet, the concentration of chloride was not dependent on the total salt deposited. Thus, the formation process and required conditions are different for different corrosion processes. In this case, the formation conditions and stage of akaganeite production are different from those of direct deposition.

The results also indicated that both the amount of salt deposited per area and the initial corrosion products influence the amount of akageneite formed. In atmospheric corrosion induced by deliquescence, the size and amount of NaCl particles determined the amount of electrolyte formed by deliquescence. A higher amount of deposited salt resulted in more electrolytes being generated. Thus, corrosion occurred over a larger area, which increased the number of places where akageneite was formed. As a result, macroscopic akaganeite could not be detected in the samples with less deposited salt.

To the best of our knowledge, the influence of the initial corrosion products on the formation of akaganeite has not previously been investigated [32]. In addition, we suggest that the initial corrosion products have two major effects. On one hand, the existence of lepidocrocite and magnetite vary the surface states and change the wettability of the surfaces. Capillary condensation may occur due to the porous nature of the corrosion products. On the other hand, lepidocrocite could participate in the corrosion process and be reduced to Fe^2+^, which is the starting material for akaganeite formation. Therefore, the presence of the initial corrosion products had a substantial impact on the formation of akaganeite.

## 4. Summary

Raman spectroscopy and XRD have been employed to study the formation mechanism of akaganeite in the initial stage of atmospheric corrosion induced by the deliquescence of NaCl particles. Several conclusions can be drawn from the results.

In high-humidity environments, the deliquescence of NaCl particles induces the formation of droplets, and atmospheric corrosion initiated quickly under the droplets. Morphologically, the electrolytes formed under these conditions were isolated and discontinuous, which guaranteed high concentrations of chloride in the droplets and sufficient oxygen supply from the surrounding environment. Additionally, these two characteristics facilitate the formation of akaganeite. In the simulated experiments, Akaganeite can form during atmospheric corrosion induced by NaCl particles within 12 h. The initial corrosion products, lepidocrocite and magnetite, increased the amount of akaganeite formed, and the amount of salt deposited played an essential role in the formation of akaganeite on the macroscopic scale.

Therefore, in marine atmospheric environments, promptly removing the deposited salts from steel surfaces and reducing salt accumulation can prevent the formation of akaganeite during deliquescence process.

## Data Availability

The original contributions presented in this study are included in the article. Further inquiries can be directed to the corresponding author.

## References

[1] Corvo F., Betancourt N., Mendoza A. (1995). The influence of airborne salinity on the atmospheric corrosion of steel. Corros. Sci..

[2] Corvo F., Haces C., Betancourt N., Maldonado L., Véleva L., Echeverria M., De Rincón O.T., Rincon A. (1997). Atmospheric corrosivity in the Caribbean area. Corros. Sci..

[3] Ma Y.T., Li Y., Wang F.H. (2008). The effect of beta-FeOOH on the corrosion behavior of low carbon steel exposed in tropic marine environment. Mater. Chem. Phys..

[4] Ma Y., Li Y., Wang F. (2009). Corrosion of low carbon steel in atmospheric environments of different chloride content. Corros. Sci..

[5] Ma Y., Li Y., Wang F. (2009). Weatherability of 09CuPCrNi steel in a tropical marine environment. Corros. Sci..

[6] Cai S., Ji H., Li M., Zhao Z., Gao Z., Zhu F. (2025). Corrosion Mechanism and Simulation of Q235 Steel in Typical Marine Atmospheric Environment. Mater. Corros..

[7] Surnam B.Y.R., Ma X., Pedrazzini S., Bilsland C., Kootab Z.S. (2025). Atmospheric Corrosion of Weathering and Mild Steels in the High Salinity Environment of Mauritius. Surf. Interface Anal..

[8] Paterlini L., Brenna A., Ceriani F., Gamba M., Ormellese M., Bolzoni F. (2024). Atmospheric Corrosion of Different Steel Types in Urban and Marine Exposure. Materials.

[9] Wu W., Zhu L., Chai P., Liu N., Song L., Liu Z., Li X. (2022). Atmospheric corrosion behavior of Nb- and Sb-added weathering steels exposed to the South China Sea. Int. J. Miner. Metall. Mater..

[10] Kamimura T., Hara S., Miyuki H., Yamashita M., Uchida H. (2006). Composition and protective ability of rust layer formed on weathering steel exposed to various environments. Corros. Sci..

[11] Yamashita M., Maeda A., Uchida H., Kamimura T., Miyuki H. (2001). Crystalline rust compositions and weathering properties of steels exposed in nation-wide atmospheres for 17 years. J. Jpn. Inst. Met..

[12] Kamimura T., Nasu S., Tazaki T., Kuzushita K., Morimoto S. (2002). Mossbauer spectroscopic study of rust formed on a weathering steel and a mild steel exposed for a long term in an industrial environment. Mater. Trans..

[13] Nishimura T., Katayama H., Noda K., Kodama T. (2000). Electrochemical behavior of rust formed on carbon steel in a wet/dry environment containing chloride ions. Corrosion.

[14] Hara S., Kamimura T., Miyuki H., Yamashita M. (2007). Taxonomy for protective ability of rust layer using its composition formed on weathering steel bridge. Corros. Sci..

[15] Morcillo M., Gonzalez-Calbet J.M., Jimenez J.A., Diaz I., Alcantara J., Chico B., Mazario-Fernandez A., Gomez-Herrero A., Llorente I., de la Fuente D. (2015). Environmental Conditions for Akaganeite Formation in Marine Atmosphere Mild Steel Corrosion Products and Its Characterization. Corrosion.

[16] Morcillo M., Chico B., Alcántara J., Díaz I., Simancas J., de la Fuente D. (2015). Atmospheric corrosion of mild steel in chloride-rich environments. Questions to be answered. Mater. Corros..

[17] Hoerle S., Mazaudier F., Dillmann P., Santarini G. (2004). Advances in understanding atmospheric corrosion of iron. II. Mechanistic modelling of wet–dry cycles. Corros. Sci..

[18] Hayashida S., Takahashi M., Deguchi H., Tsuchiya H., Hanaki K., Yamashita M., Fujimoto S. (2021). Structure of Corrosion Product Formed on Carbon Steel Covered with NiSO_4_-Added Resin Coating under Sulfuric Acid Mist Environment Containing Chloride. Mater. Trans..

[19] Zafar F., Bano H., Wahab M.F., Corvo F. (2023). Mild steel corrosion behavior in a coastal megacity relevant to China Pakistan economic corridor. NPJ Mater. Degrad..

[20] Schindelholz E., Kelly R.G. (2012). Wetting phenomena and time of wetness in atmospheric corrosion: A review. Corros. Rev..

[21] Schindelholz E., Risteen B.E., Kelly R.G. (2014). Effect of relative humidity on corrosion of steel under sea salt aerosol proxies: II. MgCl_2_, artificial seawater. J. Electrochem. Soc..

[22] Weissenrieder J., Leygraf C. (2004). In Situ Studies of Filiform Corrosion of Iron. J. Electrochem. Soc..

[23] Li S.X., Hihara L.H. (2010). Atmospheric corrosion initiation on steel from predeposited NaCl salt particles in high humidity atmospheres. Corros. Eng. Sci. Technol..

[24] Li C.L., Ma Y.T., Li Y., Wang F.H. (2010). EIS monitoring study of atmospheric corrosion under variable relative humidity. Corros. Sci..

[25] Refait P., Remazeilles C. (2007). On the formation of beta-FeOOH (akaganeite) in chloride-containing environments. Corros. Sci..

[26] Xiao H., Ye W., Song X., Ma Y., Li Y. (2017). Evolution of Akaganeite in Rust Layers Formed on Steel Submitted to Wet/Dry Cyclic Tests. Materials.

[27] Refait P., Genin J.M.R. (1997). The mechanisms of oxidation of ferrous hydroxychloride beta-Fe_2_(OH)_3_Cl in aqueous solution: The formation of akaganeite vs. goethite. Corros. Sci..

[28] Xiao H., Ye W., Song X., Ma Y., Li Y. (2017). Formation process of akaganeite in the simulated wet-dry cycles atmospheric environment. J. Mater. Sci. Technol..

[29] Li S., Hihara L.H. (2012). In situ raman spectroscopic study of NaCl particle-induced marine atmospheric corrosion of carbon steel. J. Electrochem. Soc..

[30] Forsberg J., Hedberg J., Leygraf C., Nordgren J., Duda L.C. (2010). The Initial Stages of Atmospheric Corrosion of Iron in a Saline Environment Studied with Time-Resolved In Situ X-Ray Transmission Microscopy. J. Electrochem. Soc..

[31] (1992). Corrosion of Metals and Alloys Corrosivity of Atmospheres—Classication.

[32] Xiao H., Ye W., Song X., Wang Y., Ma Y., Li Y. (2017). Determination of the key parameters involved in the formation process of akaganeite in a laboratory-simulated wet-dry cyclic process. Corros. Sci..

[33] Ohtsuka T., Tanaka S. (2015). Monitoring the development of rust layers on weathering steel using in situ Raman spectroscopy under wet-and-dry cyclic conditions. J. Solid State Electr..

[34] Cambier S.M., Verreault D., Frankel G.S. (2014). Raman investigation of anodic undermining of coated steel during environmental exposure. Corrosion.

[35] Morcillo M., Wolthuis R., Alcántara J., Chico B., Díaz I., De La Fuente D. (2016). Scanning Electron Microscopy/Micro-Raman: A Very Useful Technique for Characterizing the Morphologies of Rust Phases Formed on Carbon Steel in Atmospheric Exposures. Corrosion.

[36] Li S., Hihara L.H. (2015). A Micro-Raman Spectroscopic Study of Marine Atmospheric Corrosion of Carbon Steel: The Effect of Akaganeite. J. Electrochem. Soc..

[37] Hiller J. (1966). Phasenumwandlungen im rost. Mater. Corros..

[38] Misawa T., Asami K., Hashimoto K., Shimodaira S. (1974). The mechanism of atmospheric rusting and the protective amorphous rust on low alloy steel. Corros. Sci..

